# Effects of nitrogen application amount on nitrogen distribution and photosynthesis in tea leaves

**DOI:** 10.3389/fpls.2025.1575317

**Published:** 2025-08-01

**Authors:** Kai Li, Dong-Na Liu, Lan-Ying Li, Yuan Gao, Wan-Jun Gao, Bo-Wen Chen, Fan Luo, Yu Yao

**Affiliations:** Tea Research Institute, Tea Resources Utilization and Quality Testing Key Laboratory of Sichuan Province,Sichuan Academy of Agricultural Sciences, Chengdu, China

**Keywords:** photosynthetic nitrogen use efficiency, leaf nitrogen allocation, photosynthetic system, Camellia sinensis, nitrogen

## Abstract

Nitrogen is the most essential nutrient for plant growth and serves as a key limiting factor in overall plant development. Photosynthesis is the primary process for energy capture in the biosphere, and its effectiveness is significantly influenced by the nitrogen content and distribution within leaves. In this experiment, the yellow tea variety “Jinfeng No. 2” (hereinafter referred to as JF) and the green tea variety “Fuding Da Bai Tea” (hereinafter referred to as FD) were utilized as experimental materials. Five nitrogen levels were established to investigate the effects of varying nitrogen levels on leaf characteristics, including photosynthetic physiology, leaf nitrogen allocation, and photosynthetic nitrogen use efficiency in tea plants. The results demonstrated that the net photosynthetic rate and photosynthetic nitrogen use efficiency of both tea cultivars initially increased and then decreased with rising nitrogen levels. As nitrogen levels gradually increased, the nitrogen content in the carboxylation and electron transport systems for both tea varieties first rose and then declined. Specifically, nitrogen content in the light capture system of FD increased initially but then decreased, while in JF, it exhibited a steady increase. Additionally, nitrogen content in the structural system increased slowly, whereas that in the storage system rose significantly. With increasing nitrogen levels, the distribution ratio of leaf nitrogen in the carboxylation and electron transport systems initially increased and then decreased; in the structural system, it first decreased and subsequently stabilized, while in the storage system, it gradually increased. In the light capture system, the two varieties displayed different trends: FD’s nitrogen distribution decreased, while JF’s increased. Our results indicated that within a certain range, increasing nitrogen levels can significantly enhance the photosynthetic capacity of tea plants and improve photosynthetic nitrogen use efficiency. However, at high nitrogen levels, the reduction in nitrogen content and the proportion allocated to the photosynthetic system, along with the increase in nitrogen content and proportion allocated to non-photosynthetic systems, were the primary factors contributing to the decline in net photosynthetic rate and photosynthetic nitrogen use efficiency. By increasing the nitrogen content in the carboxylation and electron transport systems, tea plants can achieve enhanced photosynthetic capacity.

## 1 Introduction

Nitrogen, as an essential element for plant growth and development, significantly impacts crop yield and quality (Burton et al., 2024; Liang et al., 2023). Insufficient nitrogen fertilization inhibits crop growth, thereby reducing yield. Conversely, excessive nitrogen application can harm crops and increase the risk of soil acidification, agricultural non-point source pollution, and greenhouse gas emissions (Bindraban et al., 2015; Chen et al., 2022; Gu et al., 2023; Saud et al., 2022). Given the rapid expansion of the global population, the urgency to enhance crop yield has intensified, while the pollution resulting from excessive nitrogen application has become increasingly severe (Ju et al., 2006; Ma et al., 2012). Therefore, optimizing agricultural productivity while minimizing environmental impact is becoming ever more critical.

Nitrogen in plant leaves is stored in various cellular structures, with some existing as free compounds. In leaves, nitrogen can be categorized into four functional groups: respiration, photosynthesis, structural, and storage nitrogen (Ali et al., 2016). Different plants exhibit distinct nitrogen allocation strategies for each component, leading to significant variations in net photosynthetic rate and photosynthetic nitrogen use efficiency among species (Onoda et al., 2017). Structural nitrogen primarily refers to nitrogen used in the construction of cell walls and nucleic acids.Research indicates that long-lived leaves tend to invest more nitrogen in their cell walls, resulting in greater leaf mass per unit area and increased leaf toughness (Hikosaka et al., 1998; Hikosaka and Shigeno, 2009; Onoda et al., 2004). This investment enhances resistance to external changes but may reduce the overall photosynthetic rate (Qiang et al., 2023). Nitrogen storage plays an important role in plant growth, serving as a buffer mechanism that enables plants to cope with environmental changes (Li et al., 2021; Liu et al., 2018).Research on rapeseed has confirmed that simultaneous increases in photosynthetic rate and photosynthetic nitrogen use efficiency can be achieved by balancing the trade-off between stored nitrogen and active photosynthetic nitrogen (Hu et al., 2023). Photosynthetic nitrogen can be divided into three primary components: the carboxylation system, the electron transport system, and the light capture system.Generally, C_3_ plant leaves allocate more than half of their nitrogen to the photosynthetic system (Makino et al., 2003). A study involving 25 wild-type and 37 domesticated cotton varieties found that enhancing photosynthetic efficiency during cotton domestication was associated with greater nitrogen allocation to the photosynthetic apparatus (Lei et al., 2021). The capacity for CO_2_ assimilation in leaves depends on limiting factors within the processes related to carboxylation, electron transport, and light capture (Evans and Poorter, 2001). Numerical simulations indicate that optimizing nitrogen distribution among the various components of the photosynthetic apparatus can increase crop photosynthetic capacity by 60% and improve nitrogen use efficiency without additional nitrogen input (Zhu et al., 2007). Extensive research has demonstrated that plants can achieve higher net photosynthetic rates and photosynthetic nitrogen use efficiency by optimizing nitrogen allocation across different tissues under varying nitrogen application levels (Liu et al., 2023; Mu et al., 2016). However, the trade-off mechanisms of nitrogen allocation in tea leaves under different nitrogen levels remain unexplored.

The tea tree is a perennial plant characterized by its leaves. Its growth and development require significantly more nitrogen fertilizer than other crops, and both tea quality and yield are heavily influenced by nitrogen content (Bloom, 2015; Feng et al., 2014). Previous studies have primarily focused on the impacts of nitrogen levels on tea yield and quality (Lin et al., 2016, 2023; Pokharel et al., 2022; Xiao et al., 2018). Currently, tea farmers apply large quantities of nitrogen fertilizer to increase the income from tea gardens, which has led to severe environmental pollution (Lin et al., 2021; Zhu et al., 2014). Therefore, to mitigate pollution and enhance the production efficiency of tea gardens, it is essential to investigate the nitrogen use efficiency of tea trees under varying nitrogen levels. However, research on the trade-offs of nitrogen distribution in tea leaves across different nitrogen levels remains limited. This study selected the etiolated tea cultivar “JF” and the green tea cultivar “FD” as experimental subjects, with varying nitrogen levels established to: (1) investigate the impact of different nitrogen levels on nitrogen allocation in leaves of these two tea plant genotypes; (2) evaluate the relationship between distinct nitrogen allocation patterns and photosynthetic rate (Pn) and photosynthetic nitrogen use efficiency (PNUE) in tea plants. The research provides theoretical foundations and practical guidance for determining optimal nitrogen levels in tea plants, optimizing nitrogen allocation in tea leaves, and enhancing tea plantation productivity.

## 2 Materials and methods

### 2.1 Trial overview and design

This study utilized one-year-old clonal tea seedlings of the naturally occurring yellow-leaf variety (JF) and the conventional green-leaf control variety (FD) with consistent growth vigor as plant materials. The experiment was conducted in the Maohe County cultivar area pilot garden (103°22’8”N, 30°13’2”E) at the Tea Research Institute, Sichuan Academy of Agricultural Sciences. Five levels of nitrogen application were established based on urea content (N content 46%): N0 (no nitrogen fertilizer applied), N1 (220 kg hm^-^²), N2 (440 kg hm^-^², local traditional fertilization level), N3 (660 kg hm^-^²), and N4 (880 kg hm^-^²). The amounts of phosphorus and potassium fertilizer applied in each treatment were consistent with local standards. Each treatment was replicated three times, and the tea trees were transplanted into pots in April 2023. Nitrogen fertilizer was applied in three stages: 40% as base fertilizer in early October 2023, with topdressing comprising 30% for both spring and summer tea.

### 2.2 Measurement of photosynthetic characteristics

One month after the final fertilization, an open gas exchange system (Li-Cor 6800; Li-Cor Inc., Lincoln, NE, USA) was employed for measurement. To mitigate rhythmic variations in gas exchange influenced by tea leaves, measurements were conducted between 9:00 AM and 12:00 PM on a sunny, cloudless day. To further minimize the impact of leaf position and leaf age on the results, the fifth leaf from each mature bud was selected consistently. The leaf chamber parameters were set uniformly as follows:The light intensity is 1000 µmol·m^-2^·s^-1^, chamber temperature at 25°C, CO_2_ concentration at 400 µmol mol^-^¹, relative humidity controlled at 60%, and flow rate at 500 µmol s^-^¹. Prior to testing, the leaf chambers were fully filled with leaves, and the stomatal conductance (gsw) and net photosynthetic rate (P_n_) were recorded once readings stabilized (after 15-20 minutes). Subsequently, the CO_2_ response curve was established under the same environmental conditions, with CO_2_ concentration gradients in the reference chamber set to 400, 300, 200, 150, 100, 50, 400, 400, 600, 800, 1000, 1200, and 1500 µmol mol^-^¹. The A-Ci curve was calculated following the method of Long and Bernacchi (Long, 2003). Each process was repeated at least three times.

### 2.3 Sample collection and leaf morphology measurement

After determining photosynthesis, the fifth leaf of the bud was removed and transported to the laboratory, where it was placed on a black bottom plate and covered with a transparent glass layer to minimize measurement errors caused by leaf bending. Photographs were then taken, and the leaf area was calculated using Image-Pro Plus 6.0 software. Following the photography, the leaves were divided into two parts: one portion was frozen at -80°C for the determination of chlorophyll content and lipid-soluble protein nitrogen, while the other portion was dried at 105°C for 10 minutes, subsequently dried at 85°C, and weighed to determine total nitrogen content.

### 2.4 Chlorophyll determination

Fresh plant samples were taken, washed, dried, cut, and thoroughly mixed. A weight of 0.2 g from each sample was placed into a mortar, where a small amount of liquid nitrogen could be added to facilitate grinding into a powder. Subsequently, 2 mL of 95% ethanol was added to the mortar, and the mixture was ground into a homogenate. An additional 5 mL of ethanol was added, and grinding continued until the tissue became pale white. The mixture was then strained into a 25 mL brown volumetric flask. The mortar, pestle, and residue were rinsed several times with a small amount of ethanol, and the filtrate was incorporated into the volumetric flask. The ethanol was absorbed with an eyedropper, ensuring all chloroplast pigments remained on the filter paper were washed into the volumetric flask until no green color was observed on the filter paper or residue. Finally, the flask was filled with 95% ethanol to a total volume of 25 mL and shaken well. The chloroplast pigment extract was poured into a colorimetric dish with a light path of 1 cm. The absorbance was measured at 665 nm and 645 nm, with 95% ethanol serving as the blank reference.


(1) $$Chl_{a}={13.95A}_{665}-{6.88A}_{649,}$$

(2) $$Chl_{b}={24.96A}_{649}-{7.32A}_{665,}$$

(3) $$Chl_{t}=Chl_{a}+Chl_{b}$$

In **Equation 1**, Chl_a_ denotes the concentration of chlorophyll a (mg g⁻¹); in **Equation 2**, Chl_b_ denotes the concentration of chlorophyll b (mg g⁻¹); and in **Equation 3**, Chl_t_ represents the total chlorophyll content (mg g⁻¹).

### 2.5 Determination of total nitrogen content

Accurately weigh approximately 0.2 g of the mixed sample and place it into the digestion tube. Then, add a mixed catalyst consisting of 3 g of K_2_SO_4_ and 0.2 g of CuSO_4_, followed by the addition of 10 mL of concentrated sulfuric acid. Digestion was performed in a graphite digestion furnace. The digestion tube was positioned within the furnace, the exhaust hood was covered, and the exhaust gas absorption system was connected. A curve heating mode was employed for the digestion process, with specific digestion parameters set accordingly. Upon achieving a clarified state of the sample through digestion, it is ready for instrumental analysis. The fully automated Kjeldahl nitrogen analyzer (Hanon K1100F, China) integrates distillation, titration, result display, and calculation functions, enabling a comprehensive automated process.

### 2.6 Leaf functional nitrogen assimilation model

Following the methods established by Ali et al (Ali et al., 2016). and Xu et al (Xu et al., 2012), leaf nitrogen was categorized into four functional types: storage nitrogen, respiratory nitrogen, photosynthetic nitrogen, and structural nitrogen. The formulas for calculating each type of functional nitrogen are as follows:

In **Equation 4**, Leaf nitrogen content in respiratory system (N_resp_) is:


(4) $$N_{resp}=\frac{R_{t}}{33.69}$$

(5) $$R_{t}=0.015\times V_{c,max}$$

In Equation 5, R_t_ is the total leaf respiration rate (µmol CO_2_ m^−2^ s^−1^), estimated by the proportion of V_c,max_; 33.69 was the respiration rate supported by nitrogen per gram per unit time at 25°C (µmol CO_2_ g^-1^ N s^-1^).

Leaf nitrogen in storage (N_store_) is the total nitrogen content minus the remaining nitrogen content of other functional parts:


(6) $$N_{store}=N_{a}-N_{psn}-N_{resp}-N_{str}$$

In Equation 6, the nitrogen content of fat-insoluble protein (N_in-SDS_) represents the nitrogen of the structural system (Takashima et al., 2004).

### 2.7 Photosynthetic nitrogen allocation model

Referring to the research conducted by (Niinemets and Tenhunen, 1997), the photosynthetic organs in plant leaves can be categorized into three systems based on their functional differences: the electron transfer system (which includes photosynthetic electron transfer and photophosphorylation), the carboxylation system (primarily associated with Rubisco), and the light capture system (comprising PSI, PSII, LHCII, and various light-capturing pigment-protein complexes). The allocation of nitrogen content within these distinct systems of photosynthetic organs was calculated as follows:


(7) $$N_{et}=\frac{J_{max}}{8.06\times J_{mc}}$$

(8) $$N_{cb}=\frac{V_{c,max}}{6.25\times V_{cr}}$$

(9) $$N_{cl}=\frac{C_{C}}{C_{B}}$$

In Equation 7, J_mc_ represents the maximum electron transfer rate per unit of cytochrome f (Cyt f), with a value of 155.6 [electrons µmol/(µmol Cyt f·s)], and 8.06 indicates the number of μmol Cyt f contained in each gram of nitrogen converted by biomass. In Equation 8, V_cr_ denotes the specific activity of Rubisco, which refers to the CO_2_ fixation activity per unit of Rubisco protein, with a value of 20.8 [CO_2_ µmol/(g Rubisco·s)]. Additionally, Na represents the nitrogen content per unit area of leaf, and 6.25 is the coefficient for nitrogen conversion into protein. In Equation 9, C_C_ denotes the chlorophyll content of leaves (mmol/m²), C_B_ refers to the chlorophyll associated with PSI, PSII, and LHCII, with a value of 2.15 mmol/g N.

### 2.8 Data analysis

The test data were recorded using WPS 2020, and variance analysis was performed using the SPSS software package (version 27.0). The results were plotted with Origin 24.0, while data processing was conducted in R 4.2.2. A random forest regression model was constructed with the distribution content and proportion of leaf nitrogen in various leaf systems as independent variables, and the net photosynthetic rate and photosynthetic nitrogen use efficiency as dependent variables. The influence of the independent variables on the dependent variables was assessed through the increment of mean square error (IncMSE), and the significance of both the independent variables and the overall model was tested using the “rfPermute” and “A3” packages, respectively(**Supplementary Figure S1**).

## 3 Results

### 3.1 Effects of varying nitrogen levels on the photosynthetic physiology of leaves of two tea cultivars

**Figure 1:**
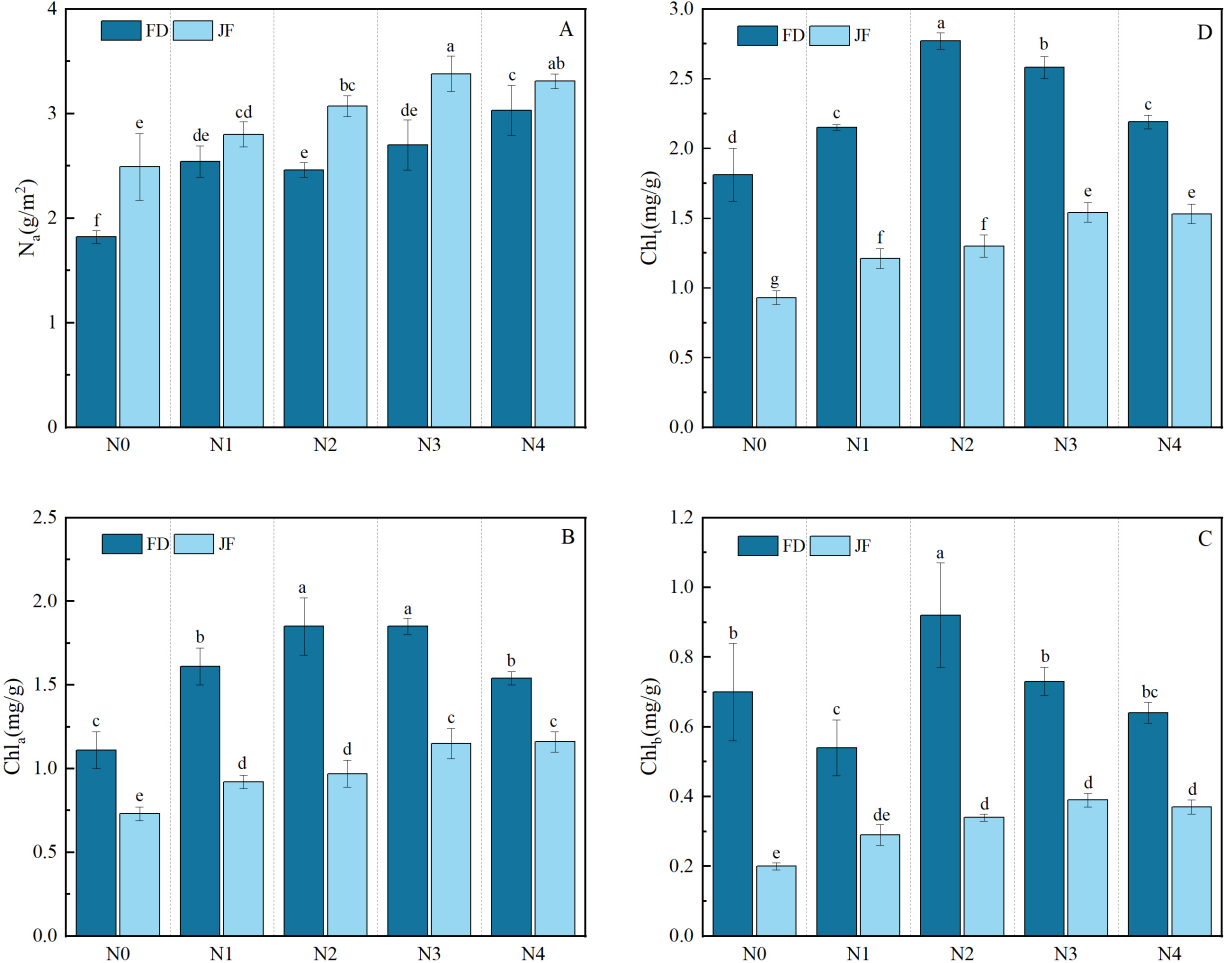
Illustrates the photosynthetic physiology of functional tea leaves under varying nitrogen levels. The longitudinal column represents the mean ± standard deviation (n = 3). Different lowercase letters in the column (a–f) signify significant differences between two varieties under various treatments for the same index (p< 0.05). The parameters measured include **(A)** nitrogen content per unit area of leaves (Na), **(B)** chlorophyll a content (Chla), **(C)** chlorophyll b content (Chlb), and **(D)** total chlorophyll content (Chlt). Values are presented as mean ± standard deviation (n = 3). The nitrogen treatments are as follows: N0: no nitrogen fertilizer applied; N1: 220 kg·hm^-^²; N2: 440 kg·hm^-^²; N3: 660 kg·hm^-^²; N4: 880 kg·hm^-^².

As illustrated in **Figure 1**, the contents of chlorophyll a, chlorophyll b, and total chlorophyll exhibited significant increases under fertilization treatments compared to non-fertilization conditions. These parameters demonstrated an initial increase followed by a subsequent decrease with escalating nitrogen application rates, while Na content showed a progressive upward trend with increasing nitrogen levels. The chlorophyll a, chlorophyll b, and total chlorophyll contents of FD reached their maximum values at the N2 level, showing respective increases of 66.67%, 31.42%, and 53.04% compared to the control group.Two-factor analysis of variance (**Table 1**) indicated that the contents of chlorophyll a, chlorophyll b, and total chlorophyll significantly differed among the treatments of various cultivars, nitrogen levels, and their interactions (P< 0.01). The effects of the cultivars on chlorophyll a, b, and total chlorophyll content were substantially greater than those of nitrogen level treatments and their interactions. The differences in Na content among various cultivars and nitrogen levels were highly significant (P< 0.01); however, the variation between cultivars and nitrogen levels was not significant.

**Table 1:** Effects of different varieties and nitrogen levels coupling on photosynthetic physiology of tea leaves.

Impact factors	Na(g/m^2^)	Chl_a_(mg/g)	Chl_b_(mg/g)	Chl_t_(mg/g)
Nitrogen(N)	30.76 ^∗∗^	38.07 ^∗∗^	8.14 ^∗∗^	67.49 ^∗∗^
Variety(V)	61.69 ^∗∗^	355.61 ^∗∗^	206.69 ^∗∗^	1022.02 ^∗∗^
N^∗^V	2.21	9.07 ^∗∗^	5.95 ^∗∗^	18.09 ^∗∗^

*P < 0.05; **P < 0.01.

### 3.2 Effects of varying nitrogen levels on the photosynthetic characteristics of leaves of two tea cultivars

**Figure 2:**
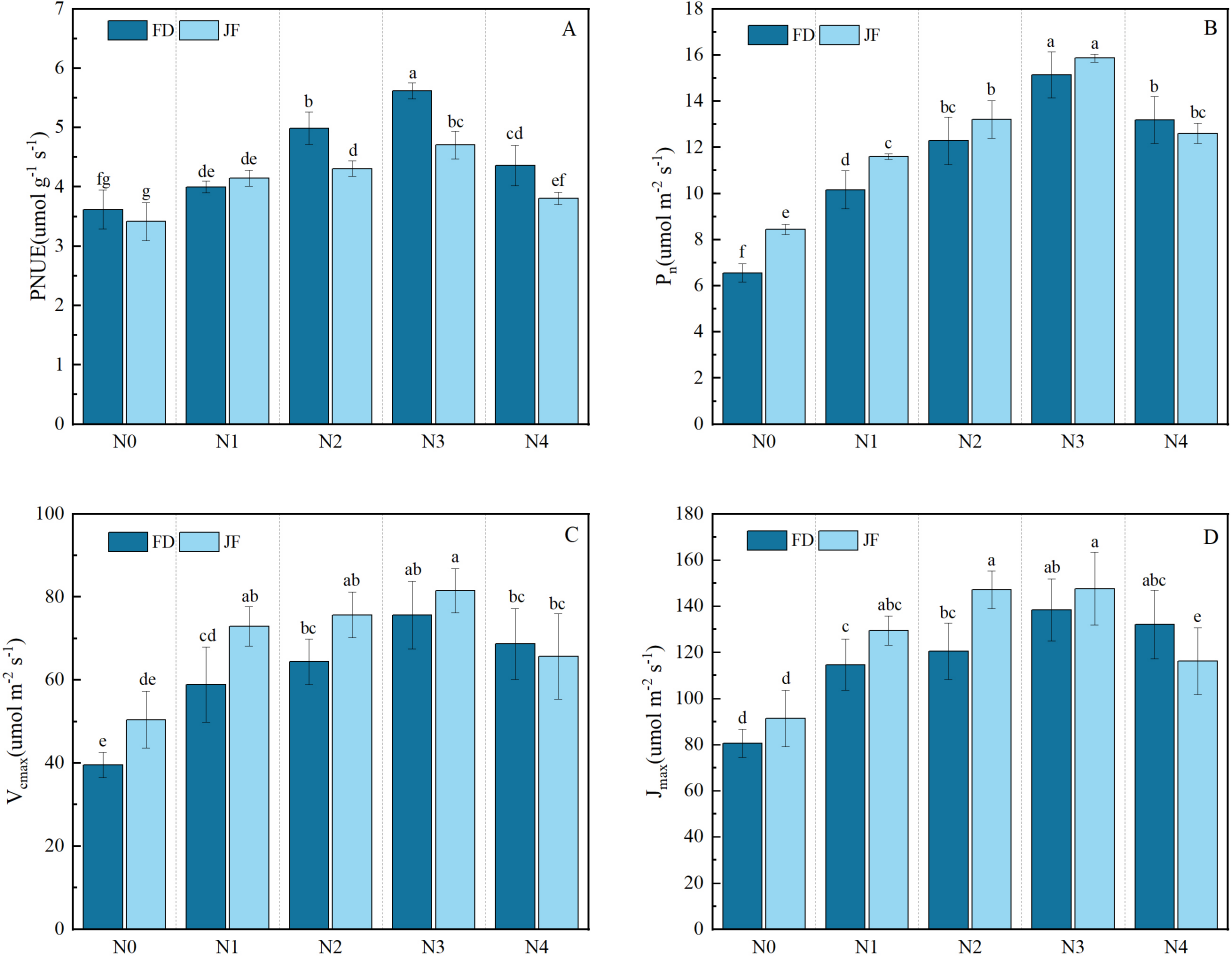
Illustrates the photosynthetic characteristics of functional tea leaves under varying nitrogen levels. The longitudinal column represents the mean ± standard deviation (n = 3). Different lowercase letters in the column (a–f) indicate significant differences between two varieties under various treatments for the same index (p< 0.05). The measured parameters include **(A)** photosynthetic nitrogen use efficiency (PNUE), **(B)** net photosynthetic rate (Asat), **(C)** maximum carboxylation rate (Vcmax), and **(D)** maximum electron transport rate (Jmax).

As illustrated in **Figure 2**, the Pn, Vcmax, and Jmax of both FD and JF exhibited identical trends in response to increasing nitrogen fertilizer application rates, reaching their peak values under the N3 treatment and subsequently demonstrating a declining trend with further elevation of nitrogen levels. Under low nitrogen conditions (N0 and N1), JF exhibited significantly higher Pn, Vcmax, and Jmax values compared to FD. However, as nitrogen levels increased, the disparity between the two cultivars gradually diminished. At the maximum nitrogen level, FD displayed higher Pn, Vcmax, and Jmax values than JF, though the difference was not statistically significant. As shown in **Figure 2**, with increasing nitrogen fertilizer application levels from N0 to N4, the PNUE of both cultivars initially increased before decreasing, reaching its maximum at N3, with increases of 55.33% and 37.83% compared to N0, respectively. No significant difference in PNUE was detected between the two cultivars at low nitrogen levels (N0 and N1); however, PNUE for FD was significantly higher than for JF at medium and high nitrogen levels. Two-factor analysis of variance (**Table 2**) indicated significant differences in PNUE across varieties, nitrogen levels, and their interactions (P< 0.01). The difference between Pn and Vcmax was highly significant under the treatments of variety and nitrogen level, but not under their interaction. Differences in Jmax were highly significant under nitrogen level treatment only, without significance under cultivar treatment or the interaction between variety and nitrogen level.

**Table 2:** Effects of different varieties and nitrogen level coupling on photosynthetic characteristics of tea leaves.

Impact factors	PNUE	P_n_	V_cmax_	J_max_
Nitrogen(N)	44.64 ∗∗	108.48 ∗∗	18.63 ∗∗	19.75 ∗∗
Variety(V)	27.48 ∗∗	11.83 ∗∗	9.28 ∗∗	4.35
N∗V	4.90 ∗∗	2.69	1.37	2.55

*P < 0.05; **P < 0.01.

### 3.3 Effects of varying nitrogen levels on nitrogen distribution in two tea cultivars

**Figure 3:**
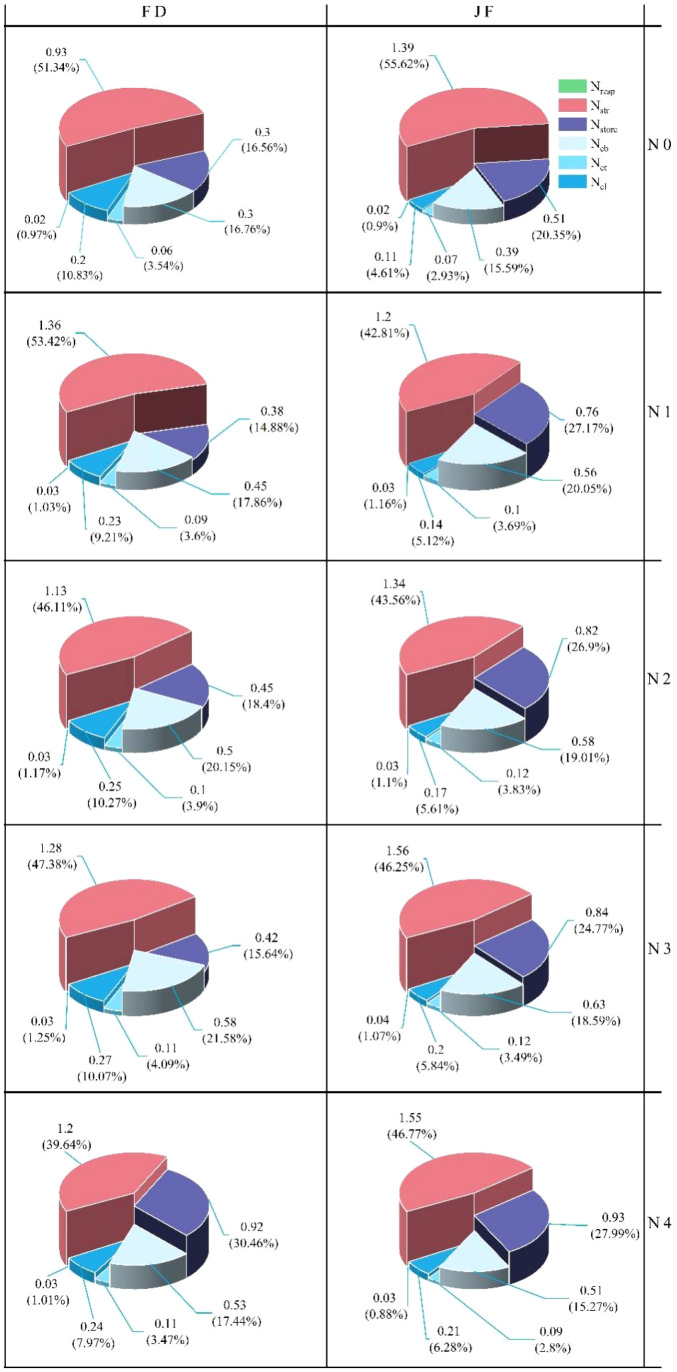
Illustrates the nitrogen distribution in tea leaves under varying nitrogen levels. Note: The value represents the absolute nitrogen content allocated to different components, while the percentage indicates the relative nitrogen content allocated among these components (n = 3). Each column corresponds to a specific variety and each row represents a treatment.

Leaf nitrogen can be categorized into four functional components: photosynthesis, respiration, storage, and structural nitrogen. As illustrated in **Figure 3**, nitrogen application levels significantly influenced the distribution of nitrogen across the components of the photosynthetic system in tea leaves. Specifically, nitrogen content increased notably in the nitrogen treatment compared to the control treatment without nitrogen application. The changes in N_cb_ content and N_et_ content were consistent across the two varieties, both displaying a trend of initially increasing and then decreasing, with the inflection point occurring at the N3 treatment. At nitrogen levels N0 to N3, the N_cb_ content and N_et_ of JF were significantly higher than those of FD. The trends of N_cl_ content and proportion in FD varied. Specifically, while the N_cl_ content of FD initially increased and then decreased with rising nitrogen levels, its distribution proportion gradually decreased. In contrast, both the N_cl_ content and distribution ratio of JF increased with elevated nitrogen levels. In general, at the same nitrogen level, the N_cl_ content and proportion of FD were significantly higher than those of JF. The nitrogen content of both FD and JF in the non-light and light systems gradually increased with rising nitrogen levels, while the distribution ratios exhibited no notable changes. Both the N_resp_ content and distribution ratio of FD and JF first increased and then decreased. For FD, the N_str_ content increased significantly initially before stabilizing, whereas the N_str_ content of JF decreased initially and then gradually increased with nitrogen fertilizer application; its distribution ratio decreased significantly at first and then stabilized. The N_store_ content and proportion for both varieties progressively increased with elevated nitrogen application rates. Under low nitrogen conditions, the nitrogen content of JF was significantly higher than that of FD; however, both varieties tended to converge under high nitrogen conditions.

### 3.4 Effects of leaf nitrogen allocation on P_n_ and PNUE

**Figure 4:**
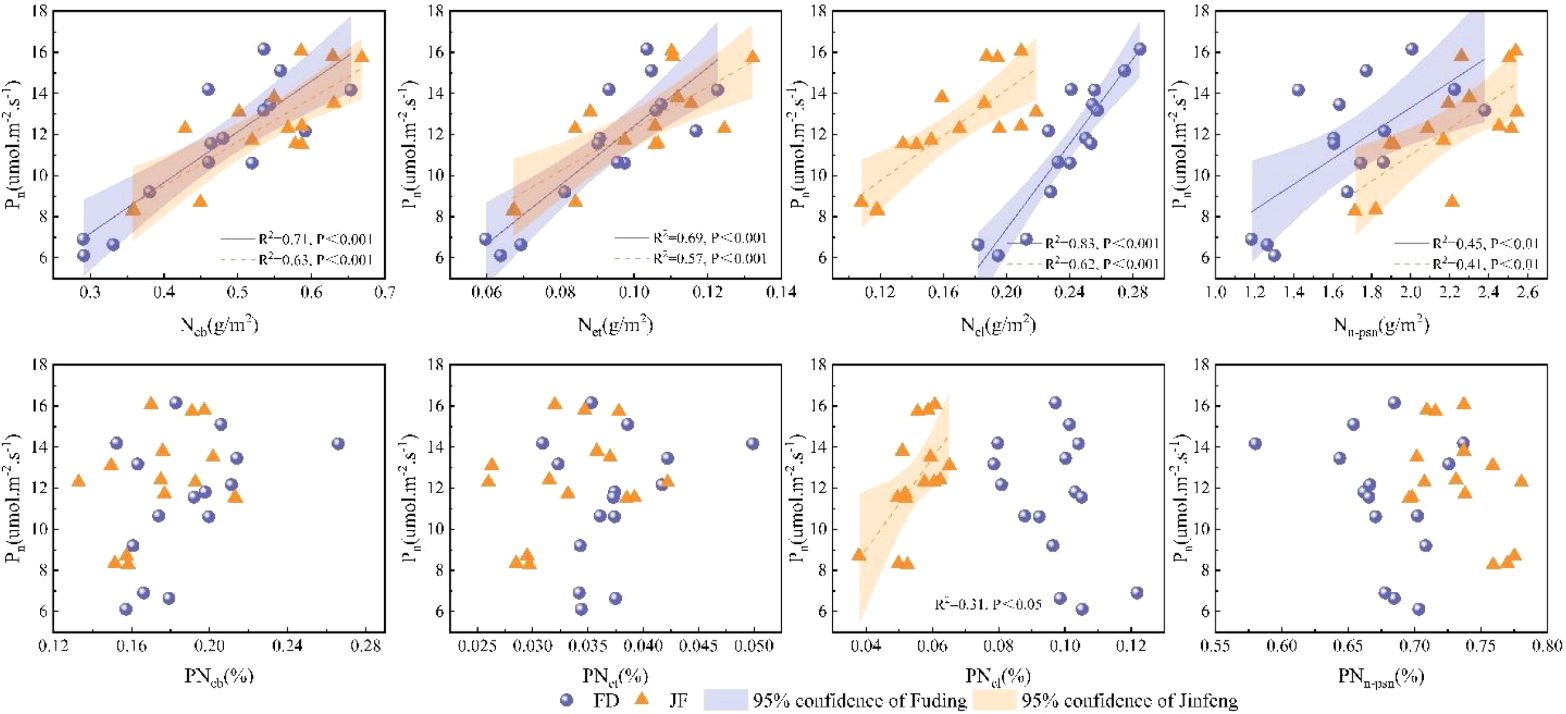
Illustrates the relationship between the carboxylation system, electron transport system, light-harvesting system, non-photosynthetic system, and the net photosynthetic rate in the functional leaves of tea trees. All data processed for each variety were analyzed collectively for correlation and fitted with linear regression.

**Figure 5:**
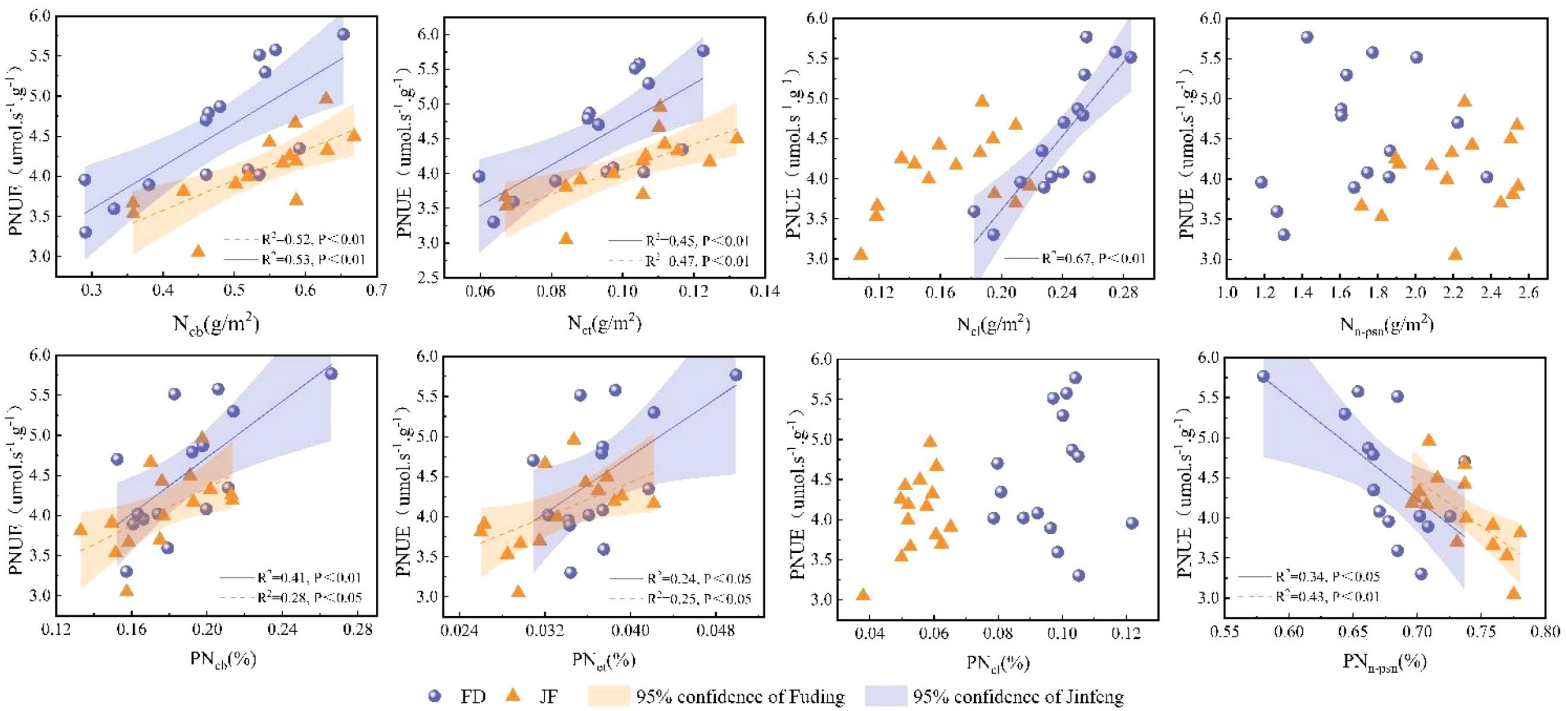
Illustrates the relationship between the carboxylation system, electron transport system, light-harvesting system, non-photosynthetic system, and photosynthetic nitrogen use efficiency in the functional leaves of tea trees. All data processed for each variety were analyzed collectively for correlation and fitted using linear regression.

Nitrogen within the leaf photosynthetic system is primarily divided into three components: the carboxylation system, the electron transport system, and the light-harvesting system.The non-light and structural systems of nitrogen encompass structural, storage, and respiratory nitrogen.Regression analysis of nitrogen content in photosynthetic and non-photosynthetic systems with respect to Pn is illustrated in **Figure 4**. Ncb, Net, and Ncl exhibit highly significant linear correlations with Pn, while Nn-psn demonstrates a significant linear correlation with Pn.Photosynthetic nitrogen use efficiency (PNUE) is a critical parameter within the leaf economic spectrum. **Figure 4** illustrates that regression analysis of photosynthetic system nitrogen content and non-light system nitrogen content showed significant linear correlations among Ncb, Net, and PNUE. However, the relationship between Ncl and PNUE varied across different varieties; it exhibited a significant linear correlation in the FD variety, whereas no notable relationship was observed in the JF variety. Additionally, the proportion of Nn-psn was negatively and linearly correlated with PNUE in both tea cultivars examined (**Figure 5**).

**Figure 6:**
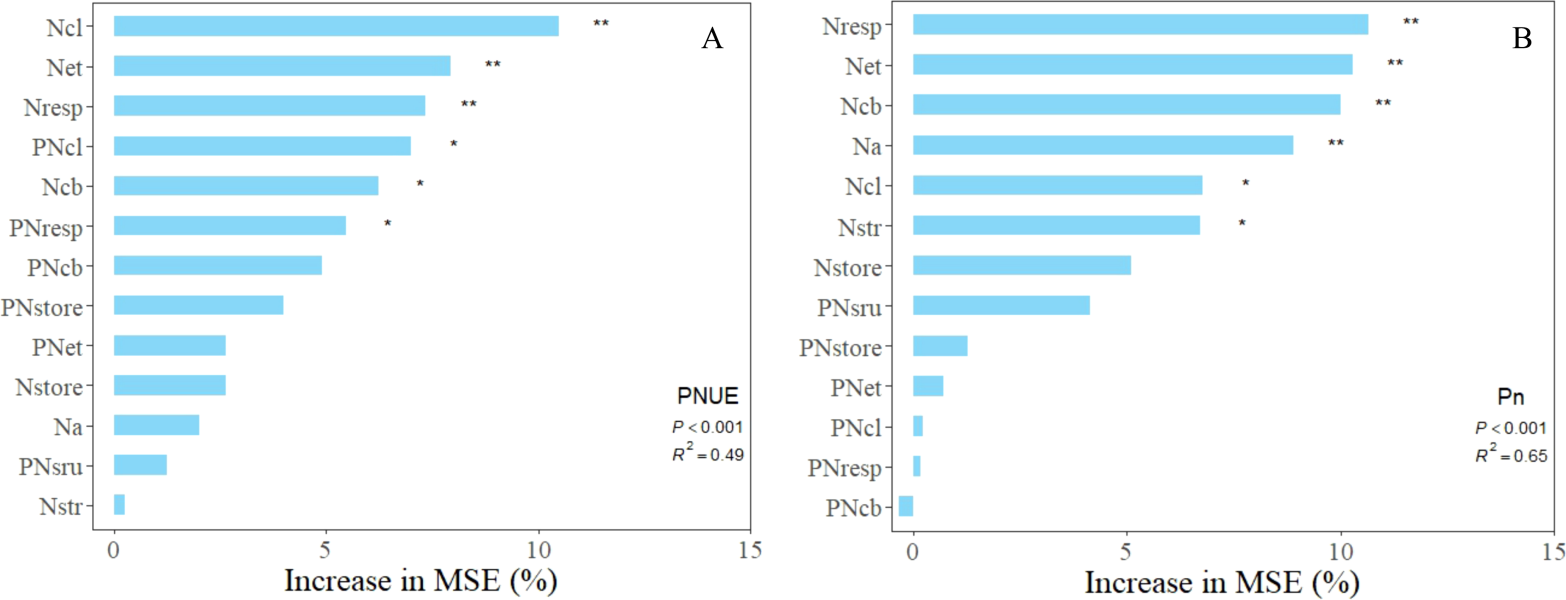
Presents the importance ranking of leaf nitrogen allocation on net photosynthetic rate **(B)** and photosynthetic nitrogen use efficiency **(A)** based on random forest regression simulation variables. ** Indicates a significant correlation at the 0.01 level, while * indicates a significant correlation at the 0.05 level.

To identify the primary drivers of photosynthetic nitrogen use efficiency (PNUE) and net photosynthetic rate (P_n_), a random forest model was developed utilizing the nitrogen distribution content and proportion of leaf nitrogen across various leaf systems. The predictors influencing changes in photosynthetic nitrogen use efficiency and net photosynthetic rate were identified through random forest analysis (see **Figure 6**). The explanation rates of the random forest models for PNUE and P_n_ were 48.9% and 65.1%, respectively (p< 0.001). The results from the random forest simulations indicate that nitrogen in the light-harvesting (N_cl_), electron transport (N_et_), and respiratory (N_resp_) systems are significant factors influencing PNUE. Similarly, N_resp_, N_et_, Na, and carboxylation (N_cb_) are critical factors impacting P_n_.

## 4 Discussion

### 4.1 Within a specific range, increasing nitrogen levels can enhance the photosynthetic capacity of the tea plant

This study revealed that, within a specific range, increasing nitrogen levels could significantly enhance the photosynthetic capacity of tea plants (Lin et al., 2016, 2023; Pokharel et al., 2022). However, excessive nitrogen levels may negatively affect the photosynthesis of tea plants, consistent with previous research (Xiao et al., 2018). A similar trend has been observed in studies on soybean and Pantoginseng (Qiang et al., 2023; Cun et al., 2023). Concurrently, as nitrogen levels increase, sodium (Na) concentrations also exhibit a gradual increase. Previous research has indicated a strong correlation between Na and photosynthetic rates (P_n_) under varying nitrogen application levels (Hou et al., 2019; Li et al., 2009) and across different species (Cheng and Fuchigami, 2000; Yamori et al., 2011). In this study, the Na concentration in the “JF” variety was significantly higher than that in “FD” across different nitrogen levels. Additionally, the net photosynthetic capacity of “JF” exceeded that of “FD” at low nitrogen levels (N0, N1, N2). However, there were no significant differences in net photosynthetic rates between the two varieties at high nitrogen levels (N3, N4). Therefore, we propose that tea tree genotypes with elevated Na concentrations exhibit higher P_n_ within a specific range, though when Na reaches a certain threshold, it no longer serves as the primary limiting factor for P_n_.

### 4.2 Reasonable regulation of nitrogen distribution in photosynthetic structure is beneficial to improve the photosynthetic capacity of tea plant

Previous studies have established a strong correlation between leaf nitrogen distribution and net photosynthetic rate. Increased nitrogen allocation to the photosynthetic system enhances both the photosynthetic capacity and growth rate of plants (Feng et al., 2009). In some natural plant species, approximately 50% of the nitrogen within leaves is dedicated to the photosynthetic apparatus (Makino et al., 2003). Nitrogen distribution within the photosynthetic apparatus can be categorized into three primary components: the carboxylation system, which includes proteins involved in carboxylation during the Calvin cycle; the electron transport system, comprised of proteins facilitating electron transport; and the light-harvesting system, which encompasses proteins responsible for light trapping in photosystems I, II, and related pigment-protein complexes. Research has indicated differing correlations between net photosynthetic rate and nitrogen content across the carboxylation, electron transport, and light-harvesting systems (Takashima et al., 2004; Gao et al., 2018). Generally, net photosynthetic rate exhibits a positive correlation with nitrogen content in both the electron transport and carboxylation systems (Hikosaka et al., 1998; Qiang et al., 2023; Zhong et al., 2019). However, studies on rice revealed that nitrogen content in the carboxylation system correlates positively with the net photosynthetic rate, whereas nitrogen content in the light-harvesting system correlates negatively, and nitrogen content in the electron transport system does not show a significant correlation (Hou et al., 2019). Consequently, the relative contribution of nitrogen content across the three components of the photosynthetic system to net photosynthetic rate varies among species. This highlights the need to identify which components could optimally enhance net photosynthetic rates. In our study, we observed that the photosynthetic rate of JF was significantly higher than that of FD under low nitrogen levels (N0, N1, N2). Notably, the nitrogen content in the carboxylation and electron transport components of JF was significantly greater than that of FD under low nitrogen conditions, while the nitrogen content in the light-harvesting system for JF was significantly lower. Further correlation analysis between the three components and net photosynthetic rate demonstrated that the carboxylation, electron transport, and light-harvesting components of both varieties were significantly positively correlated with the net photosynthetic rate. However, excessive nitrogen allocation in the light-harvesting system may trigger photoprotective responses. The nitrogen content of FD in the light-harvesting system remained elevated across different nitrogen levels, indicating a potentially greater risk of light damage for FD compared to JF. Notably, reducing, rather than increasing, the abundance of light-harvesting complexes in the photosynthetic system may prove more beneficial for overall canopy photosynthesis (Ort et al., 2010; Song et al., 2017). Therefore, we propose that strategies aimed at appropriately regulating nitrogen content in the light-harvesting system while increasing nitrogen content in the electron transport and carboxylation systems could be effective measures to enhance the photosynthetic capacity of tea trees in future research.

### 4.3 Increasing nitrogen distribution within the photosynthetic apparatus is beneficial for achieving higher photosynthetic nitrogen use efficiency

Photosynthetic nitrogen use efficiency (PNUE), defined as the photosynthetic rate per unit of nitrogen, is a key characteristic of nitrogen physiological use efficiency (Ort et al., 2010; Song et al., 2017) and exhibits a strong positive correlation with overall nitrogen physiological use efficiency. Research indicates that improving PNUE enhances both nitrogen use efficiency and crop yield (Ghannoum et al., 2005). Previous studies predominantly found that the PNUE of plants under low nitrogen treatments was higher than that under high nitrogen treatments (Liu et al., 2023; Mu et al., 2016; Zhuo et al., 2024). However, studies on crops such as rapeseed, soybean, and Notoginseng revealed that PNUE often follows a trend of initial increase, followed by a decline or stabilization as nitrogen levels rise (Qiang et al., 2023; Hu et al., 2023; Cun et al., 2023). This variability suggests that the relationship between nitrogen levels and PNUE is not constant. In our study, the PNUE of the two tea cultivars initially increased with higher nitrogen levels before subsequently decreasing. Previous research has identified the ratio of nitrogen distribution between photosynthetic and non-photosynthetic organs as a potential factor influencing variations in PNUE (Qiang et al., 2023; Flexas and Carriquí, 2020; Hikosaka, 2010). In this experiment, as nitrogen levels increased, tea plants allocated more nitrogen to the photosynthetic apparatus, resulting in a gradual increase in PNUE. Upon reaching ultra-high nitrogen levels (N4), the nitrogen capacity of the photosynthetic apparatus became saturated and began to decline, whereas the nitrogen distribution in non-photosynthetic organs did not exhibit significant changes. Consequently, we hypothesize that enhancing the nitrogen content in the electron transport system and carboxylation system, while reducing the nitrogen content in the light-harvesting system of tea plants, could significantly improve the net photosynthetic rate. This approach represents an effective strategy for future optimization of photosynthetic capacity in tea plants.

## 5 Conclusion

This study demonstrates that the photosynthetic capacity of tea plants can be significantly enhanced by increasing nitrogen levels within a specific range. At low nitrogen levels, the photosynthetic capacity of the JF cultivar surpasses that of FD due to differences in nitrogen distribution across the various components and storage structures of the photosynthetic apparatus. Increasing the nitrogen distribution ratio in the photosynthetic structures of both genotypes promotes higher photosynthetic nitrogen use efficiency (PNUE). Consequently, optimizing nitrogen distribution is expected to enhance both the net photosynthetic rate and PNUE of tea plants. These findings may provide practical guidance for optimizing nitrogen distribution in leaves, determining optimal nitrogen application levels, and efficiently breeding new tea plant varieties with high photosynthetic nitrogen use efficiency (PNUE).

## Data Availability

The original contributions presented in the study are included in the article/**Supplementary Material**. Further inquiries can be directed to the corresponding author.

## References

[B1] Ali A. A. Xu C. Rogers A. Fisher R. A. Wullschleger S. D. Massoud E. C.. (2016). A global scale mechanistic model of photosynthetic capacity (LUNA V1.0). Geoscientific Model. Dev. 9, 587–606. doi: 10.5194/gmd-9-587-2016

[B2] Bindraban P. S. Dimkpa C. Nagarajan L. Roy A. Rabbinge R. (2015). Revisiting fertilisers and fertilisation strategies for improved nutrient uptake by plants. Biol. Fertility Soils 51, 897–911. doi: 10.1007/s00374-015-1039-7

[B3] Bloom A. J. (2015). The increasing importance of distinguishing among plant nitrogen sources arnold J bloom. Curr. Opin. Plant Biol. 2015, 10–16. doi: 10.1016/j.pbi.2015.03.002, PMID: 25899331

[B4] Burton A. Häner L. L. Schaad N. Strebel S. Vuille-dit-Bille N. Bongiovani P. D. F.. (2024). Evaluating nitrogen fertilization strategies to optimize yield and grain nitrogen content in top winter wheat varieties across Switzerland. Field Crops Res. 307, 109251. doi: 10.1016/j.fcr.2024.109251

[B5] Chen C. Chu Y. Huang Q. Ding C. Zhang W. Li B.. (2022). Morphological and physiological plasticity response to low nitrogen stress in black cottonwood (Populus deltoides marsh.). J. Forestry Res. 33, 51–62. doi: 10.1007/s11676-021-01338-4

[B6] Cheng L. Fuchigami L. H. (2000). Rubisco activation state decreases with increasing nitrogen content in apple leaves. J. Exp. Bot. 51, 1687–1694. doi: 10.1093/jexbot/51.351.1687, PMID: 11053458

[B7] Cun Z. Wu H. Zhang J. Shuang S. Hong J. An T.. (2023). High nitrogen inhibits biomass and saponins accumulation in a medicinal plantPanax notoginseng. PeerJ 11, e14933. doi: 10.7717/peerj.14933, PMID: 36846464 PMC9951802

[B8] Evans J. R. Poorter H. (2001). Photosynthetic acclimation of plants to growth irradiance: the relative importance of specific leaf area and nitrogen partitioning in maximizing carbon gain. Plant Cell Environ. 24, 755–767. doi: 10.1046/j.1365-3040.2001.00724.x

[B9] Feng L. Gao M. Hou R. Hu X. Zhang L. Wan X.. (2014). Determination of quality constituents in the young leaves of albino tea cultivars. Food Chem. 155, 98–104. doi: 10.1016/j.foodchem.2014.01.044, PMID: 24594160

[B10] Feng Y. L. Lei Y. B. Wang R. F. Callaway R. M. Valiente-Banuet A. Inderjit. (2009). Evolutionary tradeoffs for nitrogen allocation to photosynthesis versus cell walls in an invasive plant. Proc. Natl. Acad. Sci. U.S.A. 106, 1853–1856. doi: 10.1073/pnas.0808434106, PMID: 19171910 PMC2644127

[B11] Flexas J. Carriquí M. (2020). Photosynthesis and photosynthetic efficiencies along the terrestrial plant’S phylogeny: lessons for improving crop photosynthesis. Plant J. 101, 964–978. doi: 10.1111/tpj.v101.4, PMID: 31833133

[B12] Gao J. Wang F. Sun J. Tian Z. Hu H. Jiang S.. (2018). Enhanced rubisco activation associated with maintenance of electron transport alleviates inhibition of photosynthesis under low nitrogen conditions in winter wheat seedlings. J. Exp. Bot. 69, 5477–5488. doi: 10.1093/jxb/ery315, PMID: 30239847

[B13] Ghannoum O. Evans J. R. Chow W. S. Andrews T. J. Conroy J. P. von Caemmerer S. (2005). Faster rubisco is the key to superior nitrogen-use efficiency in NADP-malic enzyme relative to NAD-malic enzyme C4 grasses. Plant Physiol. 137, 638–650. doi: 10.1104/pp.104.054759, PMID: 15665246 PMC1065364

[B14] Gu B. Zhang X. Lam S. K. Yu Y. van Grinsven H. J. M. Zhang S.. (2023). Cost-effective mitigation of nitrogen pollution from global croplands. Nature 613, 77–84. doi: 10.1038/s41586-022-05481-8, PMID: 36600068 PMC9842502

[B15] Hikosaka K. (2010). Mechanisms underlying interspecific variation in photosynthetic capacity across wild plant species. Plant Biotechnol. 27, 223–229. doi: 10.5511/plantbiotechnology.27.223

[B16] Hikosaka K. Hanba Y. T. Hirose T. Terashima I. (1998). Photosynthetic nitrogen-use efficiency in leaves of woody and herbaceous species. Funct. Ecol. 12, 896–905. doi: 10.1046/j.1365-2435.1998.00272.x

[B17] Hikosaka K. Shigeno A. (2009). The role of rubisco and cell walls in the interspecific variation in photosynthetic capacity. Oecologia 160, 443–451. doi: 10.1007/s00442-009-1315-z, PMID: 19288136

[B18] Hou W. Tränkner M. Lu J. Yan J. Huang S. Ren T.. (2019). Interactive effects of nitrogen and potassium on photosynthesis and photosynthetic nitrogen allocation of rice leaves. BMC Plant Biol. 19, 302. doi: 10.1186/s12870-019-1894-8, PMID: 31291890 PMC6617825

[B19] Hu W. Zhao M. Zhang S. Li Y. Dai J. Gu C.. (2023). Optimized leaf storage and photosynthetic nitrogen trade-off promote synergistic increases in photosynthetic rate and photosynthetic nitrogen use efficiency. Physiologia Plantarum 175, e14103. doi: 10.1111/ppl.v175.5, PMID: 37882267

[B20] Ju X. T. Kou C. L. Zhang F. S. Christie P. (2006). Nitrogen balance and groundwater nitrate contamination: comparison among three intensive cropping systems on the North China plain. Environ. pollut. 143, 117–125. doi: 10.1016/j.envpol.2005.11.005, PMID: 16364521

[B21] Lei Z. Wang H. Wright I. J. Zhu X. Niinemets Ü Li Z.. (2021). Enhanced photosynthetic nitrogen use efficiency and increased nitrogen allocation to photosynthetic machinery under cotton domestication. Photosynthesis Res. 150, 239–250. doi: 10.1007/s11120-021-00872-w, PMID: 34669149

[B22] Li Y. Gao Y. Xu X. Shen Q. Guo S. (2009). Light-saturated photosynthetic rate in high-nitrogen rice (Oryza sativa L.) leaves is related to chloroplastic CO2 concentration. J. Exp. Bot. 60, 2351–2360. doi: 10.1093/jxb/erp127, PMID: 19395387

[B23] Li H. Li J. Zhang X. Shi T. Chai X. Hou P.. (2021). Mesophyll conductance, photoprotective process and optimal N partitioning are essential to the maintenance of photosynthesis at N deficient condition in a wheat yellow-green mutant (Triticum aestivum L.). J. Plant Physiol. 263, 153469. doi: 10.1016/j.jplph.2021.153469, PMID: 34252704

[B24] Liang G. Hua Y. Chen H. Luo J. Xiang H. Song H.. (2023). Increased nitrogen use efficiency via amino acid remobilization from source to sink organs in brassica napus. Crop J. 11, 119–131. doi: 10.1016/j.cj.2022.05.011

[B25] Lin Z. Chen C. Zhao S. Liu Y. Zhong Q. Ruan Q.. (2023). Molecular and physiological mechanisms of tea (Camellia sinensis (L.) O. Kuntze) leaf and root in response to nitrogen deficiency. BMC Genomics 24, 27. doi: 10.1186/s12864-023-09112-y, PMID: 36650452 PMC9847173

[B26] Lin Z. Chen C. Zhong Q. Ruan Q. Chen Z. You X.. (2021). The GC-TOF/MS-based metabolomic analysis reveals altered metabolic profiles in nitrogen-deficient leaves and roots of tea plants (Camellia sinensis). BMC Plant Biol. 21, 506. doi: 10.1186/s12870-021-03285-y, PMID: 34727870 PMC8561955

[B27] Lin Z. Zhong Q. Chen C. Ruan Q. Chen Z. You X. (2016). Carbon dioxide assimilation and photosynthetic electron transport of tea leaves under nitrogen deficiency. Botanical Stud. 57. doi: 10.1186/s40529-016-0152-8, PMID: 28597447 PMC5432892

[B28] Liu T. Ren T. White P. J. Cong R. Lu J. (2018). Storage nitrogen co-ordinates leaf expansion and photosynthetic capacity in winter oilseed rape. J. Exp. Bot. 69, 2995–3007. doi: 10.1093/jxb/ery134, PMID: 29669007 PMC5972566

[B29] Liu J. Zhang K. Bi J. Yu X. Luo L. Hu L. (2023). Mesophyll conductance and N allocation co-explained the variation in photosynthesis in two canola genotypes under contrasting nitrogen supply. Front. Plant Sci. 14, 1171331. doi: 10.3389/fpls.2023.1171331, PMID: 37223789 PMC10202220

[B30] Long S. P. (2003). Gas exchange measurements, what can they tell us about the underlying limitations to photosynthesis? Procedures and sources of error. J. Exp. Bot. 54, 2393–2401. doi: 10.1093/jxb/erg262, PMID: 14512377

[B31] Ma L. Velthof G. L. Wang F. H. Qin W. Zhang W. F. Liu Z.. (2012). Nitrogen and phosphorus use efficiencies and losses in the food chain in China at regional scales in 1980 and 2005. Sci. Total Environ. 434, 51–61. doi: 10.1016/j.scitotenv.2012.03.028, PMID: 22542299

[B32] Makino A. Sakuma H. Sudo E. Mae T. (2003). Differences between maize and rice in N-use efficiency for photosynthesis and protein allocation. Plant Cell Physiol. 44, 952–956. doi: 10.1093/pcp/pcg113, PMID: 14519777

[B33] Mu X. Chen Q. Chen F. Yuan L. Mi G. (2016). Within-leaf nitrogen allocation in adaptation to low nitrogen supply in maize during grain-filling stage. Front. Plant Sci. 7, 194737. doi: 10.3389/fpls.2016.00699, PMID: 27252716 PMC4877366

[B34] Niinemets Ü Tenhunen J. D. (1997). A model separating leaf structural and physiological effects on carbon gain along light gradients for the shade-tolerant species acer saccharum. Plant Cell Environ. 20, 845–866. doi: 10.1046/j.1365-3040.1997.d01-133.x

[B35] Onoda Y. Hikosaka K. Hirose T. (2004). Allocation of nitrogen to cell walls decreases photosynthetic nitrogen-use efficiency. Funct. Ecol. 18, 419–425. doi: 10.1111/j.0269-8463.2004.00847.x

[B36] Onoda Y. Wright I. J. Evans J. R. Hikosaka K. Kitajima K. Niinemets Ü. (2017). Physiological and structural tradeoffs underlying the leaf economics spectrum. New Phytol. 214, 1447–1463. doi: 10.1111/nph.2017.214.issue-4, PMID: 28295374

[B37] Ort D. R. Zhu X. Melis A. (2010). Optimizing antenna size to maximize photosynthetic efficiency. Plant Physiol 155, 79–85. doi: 10.1104/pp.110.165886, PMID: 21078863 PMC3014228

[B38] Pokharel S. S. Zhong Y. Changning L. Shen F. Likun L. Parajulee M. N.. (2022). Influence of reduced N-fertilizer application on foliar chemicals and functional qualities of tea plants under toxoptera aurantii infestation. BMC Plant Biol. 22, 166. doi: 10.1186/s12870-022-03533-9, PMID: 35366797 PMC8976352

[B39] Qiang B. Zhou W. Zhong X. Fu C. Cao L. Zhang Y.. (2023). Effect of nitrogen application levels on photosynthetic nitrogen distribution and use efficiency in soybean seedling leaves. J. Plant Physiol. 287, 154051. doi: 10.1016/j.jplph.2023.154051, PMID: 37481898

[B40] Saud S. Wang D. Fahad S. (2022). Improved nitrogen use efficiency and greenhouse gas emissions in agricultural soils as producers of biological nitrification inhibitors. Front. Plant Sci. 13, 854195. doi: 10.3389/fpls.2022.854195, PMID: 35432390 PMC9011059

[B41] Song Q. Wang Y. Qu M. Ort D. R. Zhu X. G. (2017). The impact of modifying photosystem antenna size on canopy photosynthetic efficiency—Development of a new canopy photosynthesis model scaling from metabolism to canopy level processes. Plant Cell Environ. 40, 2946–2957. doi: 10.1111/pce.v40.12, PMID: 28755407 PMC5724688

[B42] Takashima T. Hikosaka K. Hirose T. (2004). Photosynthesis or persistence: nitrogen allocation in leaves of evergreen and deciduous quercus species. Plant Cell Environ. 27, 1047–1054. doi: 10.1111/j.1365-3040.2004.01209.x

[B43] Xiao F. Yang Z. Huang H. Yang F. Zhu L. Han D. (2018). Nitrogen fertilization in soil affects physiological characteristics and quality of green tea leaves. Hortscience 53, 715–722. doi: 10.21273/HORTSCI12897-18

[B44] Xu G. Fan X. Miller A. J. (2012). Plant nitrogen assimilation and use efficiency. Annu. Rev. Plant Biol. 63, 153–182. doi: 10.1146/annurev-arplant-042811-105532, PMID: 22224450

[B45] Yamori W. Nagai T. Makino A. (2011). The rate-limiting step for CO2 assimilation at different temperatures is influenced by the leaf nitrogen content in several C3 crop species. Plant Cell Environ. 34, 764–777. doi: 10.1111/j.1365-3040.2011.02280.x, PMID: 21241332

[B46] Zhong C. Jian S. F. Huang J. Jin Q. Y. Cao X. C. (2019). Trade-off of within-leaf nitrogen allocation between photosynthetic nitrogen-use efficiency and water deficit stress acclimation in rice (Oryza sativa L.). Plant Physiol. Biochem. 135, 41–50. doi: 10.1016/j.plaphy.2018.11.021, PMID: 30500517

[B47] Zhu X. de Sturler E. Long S. P. (2007). Optimizing the distribution of resources between enzymes of carbon metabolism can dramatically increase photosynthetic rate: A numerical simulation using an evolutionary algorithm. Plant Physiol. 145, 513–526. doi: 10.1104/pp.107.103713, PMID: 17720759 PMC2048738

[B48] Zhu T. Zhang J. Meng T. Zhang Y. Yang J. Müller C.. (2014). Tea plantation destroys soil retention of NO3– and increases N2O emissions in subtropical China. Soil Biol. Biochem. 73, 106–114. doi: 10.1016/j.soilbio.2014.02.016

[B49] Zhuo H. Liu X. Luo S. Ou X. Rong X. Yang L.. (2024). Physiological changes underlying increased photosynthetic-nitrogen use efficiency in response to low-nitrogen conditions in brassica napus L. Ind. Crops Products 211, 118240. doi: 10.1016/j.indcrop.2024.118240

