# Supplementary material for: Effects of nitrogen application amount on nitrogen distribution and photosynthesis in tea leaves
Source: Front Plant Sci. 2025 Aug 1;16:1575317. doi: 10.3389/fpls.2025.1575317 (PMC12354480; doi:10.3389/fpls.2025.1575317)
Supplement: Supplementary file 1 [file DataSheet1.pdf]

```r
env <- read.csv("sjsl.csv")library("randomForest")
set.seed(123)
env_forest <- randomForest(PNUE~., data = env, importance = TRUE, ntree
= 500, nPerm = 1000)
env_forest

importance_env.scale <- data.frame(importance(env_forest, scale = TRUE),
check.names = FALSE)
importance_env.scale

importance_env.scale <-
importance_env.scale[order(importance_env.scale$`%IncMSE`, decreasing
= TRUE), ]

library(ggplot2)
importance_env.scale$env_name <- rownames(importance_env.scale)
importance_env.scale$env_name <- factor(importance_env.scale$env_name,
                                        levels =
importance_env.scale$env_name)
ggplot(importance_env.scale, aes(env_name, `%IncMSE`))+
  geom_col(width = 0.5, fill = 'skyblue', color = NA)+
  labs(title = NULL, x = NULL, y = 'Increase in MSE(%)', fill = NULL)+
  theme(panel.grid = element_blank(), panel.background = element_blank(),
axis.line = element_line(colour = 'black')) +
  theme(axis.text.x = element_text(angle = 45, hjust = 1)) +
  scale_y_continuous(expand = c(0, 0), limit = c(0, 16))


library(rfPermute)
set.seed(123)
env_rfP <- rfPermute(PNUE~., data = env, importance = TRUE, ntree = 500,
                     nrep = 1000, num.cores = 1)

importance_env.scale <- data.frame(importance(env_rfP, scale = TRUE),
check.names = FALSE)
importance_env.scale

importance_env.scale.pval <- (env_rfP$pval)[ , , 2]
importance_env.scale.pval

importance_env.scale <-
importance_env.scale[order(importance_env.scale$'%IncMSE',
                                                 decreasing = FALSE), ]
```

```
library(ggplot2)
importance_env.scale$env_name <- rownames(importance_env.scale)
importance_env.scale$env_name <- factor(importance_env.scale$env_name,
levels = importance_env.scale$env_name)
p <- ggplot() +
  geom_col(data = importance_env.scale, aes(x = env_name, y = `%IncMSE`),
width = 0.5, fill = "skyblue", color = NA) +
  labs(title = NULL, x = NULL, y = 'Increase in MSE (%)', fill = NULL)
+
  theme(panel.grid = element_blank(), panel.background = element_blank(),
axis.line = element_line(colour = 'black')) +
  theme(axis.text.x = element_text(angle = 45, hjust = 1)) +
  scale_y_continuous(expand = c(0, 0), limit = c(0, 15))
p
```

```
for (env in rownames(importance_env.scale)) {
  if (importance_env.scale[env, '%IncMSE.pval'] >= 0.05) {
    importance_env.scale[env, '%IncMSE.sig'] <- ''
  } else if (importance_env.scale[env, '%IncMSE.pval'] >= 0.01 &
importance_env.scale[env, '%IncMSE.pval'] < 0.05) {
    importance_env.scale[env, '%IncMSE.sig'] <- '*'
  } else if (importance_env.scale[env, '%IncMSE.pval'] >= 0.001 &
importance_env.scale[env, '%IncMSE.pval'] < 0.01) {
    importance_env.scale[env, '%IncMSE.sig'] <- '**'
  } else if (importance_env.scale[env, '%IncMSE.pval'] < 0.001) {
    importance_env.scale[env, '%IncMSE.sig'] <- '***'
  }
}
p <- p +
  geom_text(data = importance_env.scale, aes(x = env_name, y = `%IncMSE`,
label = `%IncMSE.sig`), nudge_y = 1)+
  theme_bw()+
  theme(panel.grid = element_blank())+
  coord_flip()
p
```

```
library(A3)

set.seed(123)
```

```
env_forest.pval <- a3(PNUE~., data = env, model.fn = randomForest, p.acc
= 0.001, model.args = list(importance = TRUE, ntree = 500))
env_forest.pval


p <- p +
  annotate('text',label = 'PNUE',x=1.4,y=14,size=4)+
  annotate('text', label = 'italic(P) < 0.001', x = 1.0, y = 14, size =
3, parse = TRUE) +
  annotate('text', label = sprintf('italic(R^2) == %.2f', 90.63), x = 0.7,
y = 14, size = 3, parse = TRUE)
p
```
